# The effect of visceral fat on the hemodilution effect of serum carcinoembryonic antigen in Korean population

**DOI:** 10.1371/journal.pone.0225649

**Published:** 2019-12-02

**Authors:** Youn-Joon Jung, Seung-Su Han

**Affiliations:** Department of Obstetrics and Gynecology, Chung-Ang University College of Medicine, Seoul, Korea; San Raffaele Roma Open University, ITALY

## Abstract

**Objective:**

To investigate the relationship between visceral fat and the hemodilution effect of carcinoembryonic antigen in both sexes.

**Methods:**

A total of 15,340 females and 20,024 males who visited the health promotion center at Chung-Ang University Hospital from 2011 to 2014 were retrospectively collected. Correlation analysis and chi-square test for linear by linear association were used to determine the correlation between carcinoembryonic antigen concentration, carcinoembryonic antigen mass and visceral fat. Multivariable linear regression analysis was used to calculate the mean of carcinoembryonic antigen concentration and the mean of carcinoembryonic antigen mass, reflecting age, aspartate aminotransferase, alanine aminotransferase, creatinine, body fat percentage, body mass index, lean body mass and waist circumference as confounding variables.

**Results:**

Higher body mass index was related with lower carcinoembryonic antigen concentration in men (r = -0.019, P = 0.019), but higher carcinoembryonic antigen concentration in women (r = 0.084, P<0.001). Average of waist circumference for male is greater than that of female (P<0.01). Average of body fat percentage for male is lesser than that of female (P<0.01). Male lean body mass mean is larger than that of women (P<0.01). Increased waist circumference was significantly associated with higher carcinoembryonic antigen mass in both female and male (P<0.001 for trend). Postmenopausal women might be more likely to have increased carcinoembryonic antigen mass and carcinoembryonic antigen concentration (P<0.001 for trend).

**Conclusions:**

This study suggests that visceral fat may increase total amount of CEA in the body. Visceral fat should be taken into account when evaluating serum CEA levels in both sexes.

## Introduction

Cancer is the second leading cause of death globally and accounted for 8.8 million death in 2015 according to the World Health Organization [1]. However, early diagnosis of cancer can improve the survival rate. Because tumor markers are important in early diagnosis of cancer, precise measurement and criteria of tumor marker are needed.

Obesity has been identified as a risk factor for several cancers. Previous studies have shown the correlation between cancer and obesity [2–4]. In addition, increased visceral fat in obesity is related to metabolic syndrome, and metabolic syndrome is also a risk factor for cancer [5]. Therefore, early diagnosis through screening test is even more important for obese people, and accurate tumor marker standards are emphasized. However, obese people may be difficult to diagnose early due to the hemodilution effect, which refers to diluted tumor marker concentrations as a result of increased plasma volume secondary to obesity [6–8].

In our previous study, CA 125 and CA 19–9 followed the hemodilution effect, but Carcinoembryonic antigen (CEA) and α-fetoprotein (AFP) showed positive correlations with body mass index (BMI) in women [9]. CEA has been shown to increase not only in cancer but also in the metabolic syndrome, which is closely associated with obesity [10, 11]. In addition, CEA concentration increases with increasing visceral fat in women [12]. These findings suggest that visceral fat which is a common risk factor for cancer and metabolic syndrome that may be associated with elevated CEA. This association will affect cancer screening using CEA in obese people. Therefore, we investigated the relationship between serum CEA concentration and visceral fat in Korean women and men.

## Methods

### Patients and clinical variables

This retrospective study was approved by the Institutional Review Board of the Chung-Ang university hospital (approval No. 2016–1639). The electronic medical records of 15,340 women and 20,024 men who visited the Health Promotion Center at Chung-Ang University Hospital for routine examination from 2011 to 2014 were reviewed for the retrospective analysis. Patients underwent screening for tumor markers involving CEA. Serum creatinine, serum alanine aminotransferase (ALT) and serum aspartate aminotransferase (AST) were measured to monitor the renal and liver function, which may affect the metabolism of tumor markers. The records of 14,374 women and 16,953 men were obtained after excluding those with abnormal data (CEA greater than 5.0 ng/ml) to minimize the effects of unknown cancers and benign conditions that affect tumor marker levels, such as pro-inflammatory conditions. CEA concentrations were measured as ng/ml. The weight and height of patients were measured directly. BMI was calculated by dividing weight in kg by the square of the height in meters, and patients were stratified by the WHO recommendations for Asian populations for international comparison. Patients were categorized according to BMI less than 18.5, 18.5 to less than 23, 23 to less than 27.5 and 27.5 or greater. According to metabolic syndrome diagnostic criteria of the National Cholesterol Education Program (NCEP) Adult Treatment Panel III (ATP III), waist circumference (WC) was divided into two groups based on 80cm for females and 90cm for males. Women over 50 years old were classified as menopausal [13–15]. Estimated body surface area (BSA) was calculated as (bodyweight)^0.425^ × (height)^0.72^ × 0.007184 [16]. Estimated plasma volume (in liters) was calculated from BSA×1.670 [17]. Estimated Lean body mass (LBM) was calculated as (0.29569 × weight) + (0.41813 × height)– 43.2933 for women, and (0.32810 × weight) + (0.33929 × height)– 29.5336 for men [18]. Tumor marker mass (in micro-grams) was defined as the total amount of tumor marker proteins in circulation at the time of the examination and was calculated as serum tumor marker concentration times total circulating plasma volume.

### Statistical analysis

Pearson correlation analysis was used to examine the association between BMI and tumor marker concentration. Serum CEA concentrations not exhibiting a normal distribution were analyzed with continuous terms after logarithmic transformation and were back-transformed to ease interpretation. Multivariable linear regression analysis was used to calculate the mean of CEA concentration and the mean of CEA mass, considering age, AST, ALT, creatinine, body fat percentage, BMI, LBM and WC as confounding variables. Trends in serum tumor marker concentrations and tumor marker masses were tested for across BMI categories across BMI categories, waist circumference categories and menopausal grouping by chi-square test for linear by linear association. SPSS 19.0 was used for statistical analysis and associations with P<0.05 were considered statistically significant.

## Results

Table 1 lists the demographic and physical characteristics, plasma volume, serum CEA concentration, CEA mass, AST, ALT, and creatinine. Male subjects had a higher age, BMI, WC, LBM, CEA, AST, ALT, and creatinine level, but lower body fat percentage (P<0.01).

**Table 1:** Clinical characteristics of 14,374 female and 16,942 male undergoing tumor marker screening.

Variable	Total	Female	Male	P value
Age (years)	44.20 ± 10.82 (12.00–91.00)	44.01 ± 11.19 (16.00–90.00)	44.37 ± 10.49 (12.00–91.00)	<0.01
BMI (kg/m^2^)^†^	23.61 ± 3.40 (11.77–48.66)	22.27 ± 3.31 (11.77–47.91)	24.76 ± 3.04 (15.09–48.66)	<0.01
<18.5	17.61 ± 0.73 (11.77–18.50)	17.61 ± 0.75 (11.77–18.50)	17.64 ± 0.73 (15.09–18.50)	0.669
18.5–23.0	21.08 ± 1.24 (18.50–23.00)	20.81 ± 1.23 (18.50–23.00)	21.53 ± 1.11 (18.50–23.00)	<0.01
23.0–27.5	24.94 ± 1.23 (23.00–27.50)	24.73 ± 1.21 (23.00–27.50)	25.04 ± 1.23 (23.00–27.50)	<0.01
≥27.5	29.76 ± 2.37 (27.50–48.66)	30.14 ± 2.74 (27.50–47.91)	29.63 ± 2.20 (27.50–48.66)	<0.01
Body fat percentage(%)	26.85 ± 6.68 (3.00–53.80)	30.60 ± 6.09 (3.00–53.80)	23.67 ± 5.38 (3.00–51.20)	<0.01
Waist circumference(cm)	82.45 ± 9.51 (45.00–145.00)	77.41 ± 8.71 (45.00–130.00)	86.71 ± 7.95 (52.00–145.00)	<0.01
Lean body mass	47.23 ± 7.76 (26.91–81.58)	40.32 ± 3.90 (26.81–66.08)	53.09 ± 4.84 (33.23–81.58)	<0.01
Body surface area(m^2^)	1.69 ± 0.19 (1.11–2.59)	1.54 ± 0.11 (1.11–2.29)	1.82 ± 0.14 (1.27–2.59)	<0.01
Plasma volume(liter)	2.82 ± 0.31 (1.85–4.32)	2.57 ± 0.19 (1.85–3.83)	3.03 ± 0.23 (2.11–4.32)	<0.01
CEA(ng/mL)	1.73 ± 0.97 (0.20–5.00)	1.42 ± 0.83 (0.20–4.99)	2.00 ± 1.01 (0.20–5.00)	<0.01
CEA mass(μg)	4.94 ± 2.93 (0.43–20.81)	3.63 ± 2.14 (1.85–3.83)	6.04 ± 3.06 (0.52–20.81)	<0.01
ALT(IU/L)	25.85 ± 21.69 (2.00–662.00)	18.53 ± 15.20 (2.00–534.00)	32.06 ± 24.28 (3.00–662.00)	<0.01
AST(IU/L)	25.98 ± 13.33 (6.00–483.00)	23.02 ± 11.15 (9.00–483.00)	28.49 ± 14.47 (6.00–400.00)	<0.01
Creatinine(mg/dL)	0.81 ± 0.19 (0.32–9.09)	0.67 ± 0.14 (0.32–9.09)	0.93 ± 0.14 (0.43–6.10)	<0.01

BMI, body mass index; CEA, carcinoembryonic antigen; ALT, alanine aminotransferase; AST, aspartate aminotransferase.

Data were presented as mean ± standard deviation (SD) according to a normal distribution.

P-values were calculated using the independent student`s T-test.

^†^Patients were stratified by the WHO recommendations for Asian population for international comparison.

Table 2 shows the distribution of subjects according to BMI classification and waist circumference in females and males, and the distribution of subjects according to menopause in females. As a percentage, the proportion of obesity in male subjects was significantly higher than that in female subjects. The proportion of WC exceeding the criteria in female subjects (35.82%) was significantly higher than that in male subjects (33.19%). A total of 26.97% of female were post-menopausal.

**Table 2:** Population and percentage of study subjects in each group.

Variable	Total:31,327(%)	Female:14,374(%)	Male:16,953(%)	P value
BMI				
<18.5	1,448(4.62)	1,281(8.91)	167(0.99)	<0.001
18.5–23.0	12,745(40.68)	8,108(56.41)	4,637(27.35)	
23.0–27.5	13,349(42.61)	3,975(27.65)	9,374(55.29)	
≥27.5	3,785(12.08)	1,010(7.03)	2,775(16.37)	
Waist circumference^†^				
<criteria	-	9,225(64.18)	11,326(66.81)	<0.001
≥criteria		5,149(35.82)	5,627(33.19)	
Menopause				
Pre-menopause	-	10,497(73.03)	-	-
Post-menopause		3,877(26.97)		

BMI, body mass index.

Data are n (%).

P-values were calculated using the chi-square test.

^†^According to diagnostic criteria of metabolic syndrome by National Cholesterol Education Program (NCEP) Adult Treatment Panel III (ATPIII), waist circumference was divided into two groups based on 80cm for female and 90cm for male.

**Fig 1:**
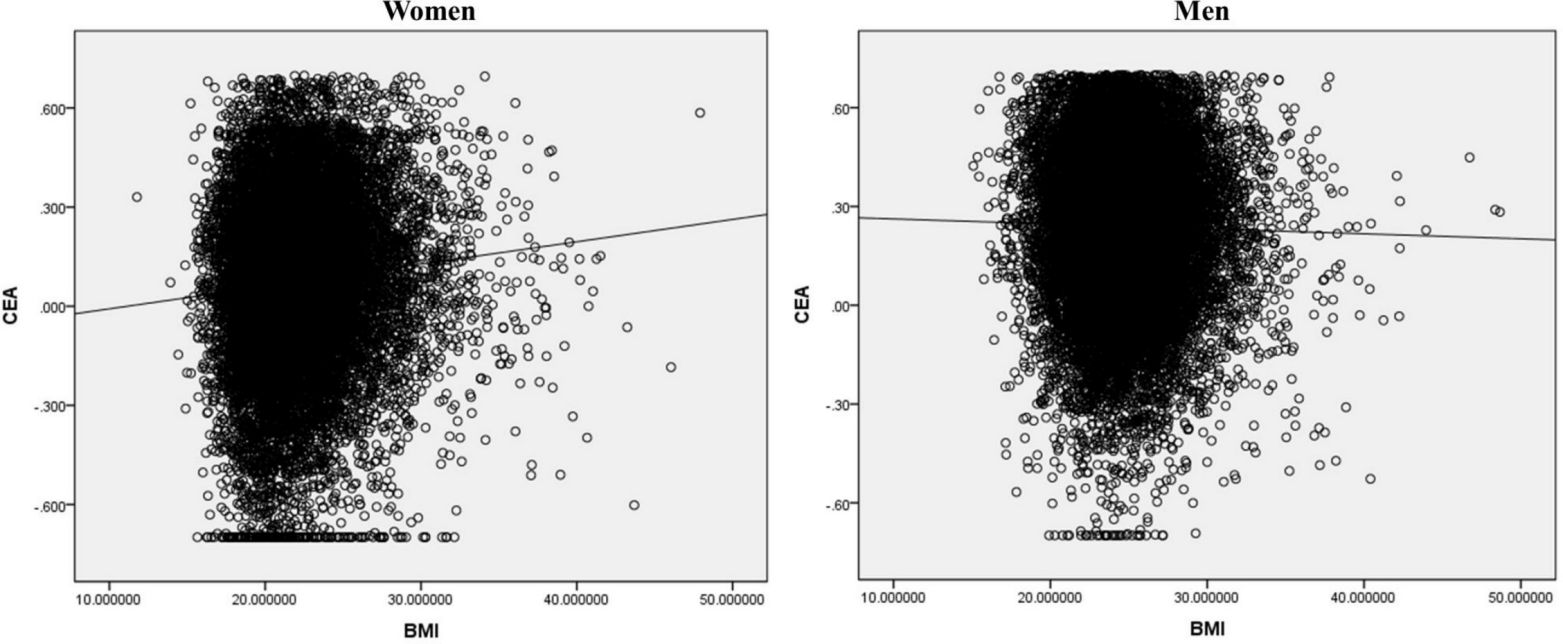
Relationship between serum CEA concentration and BMI in each gender. Higher BMI was related with higher CEA concentration in women (r = 0.084, P<0.001) but lower CEA in men (r = -0.019, P = 0.013).

Higher BMI was related with higher CEA concentration in women (r = 0.084, P<0.001) but lower CEA concentration in men (r = -0.019, P = 0.013) (Fig 1).

Plasma volume was significantly increased with higher BMI in both sexes (P for trend <0.01). After controlling for age, waist circumference, BMI, AST, ALT and creatinine, the relations between serum CEA concentration, CEA mass and BMI, waist circumference, menopause were analyzed (Tables 3–5). Table 3 shows that higher CEA concentration (P for trend <0.01) and CEA mass (P for trend <0.01) were associated with higher BMI in females. Increased BMI seems to decrease CEA concentration in males, but this is not statistically significant (P = 0.074). In Table 4, greater CEA concentration (P for trend <0.01) and CEA mass (P for trend <0.01) were significantly associated with higher waist circumference in females. Greater CEA mass (P for trend <0.01) was related to higher waist circumference, but CEA concentration (P = 0.595) did not change according to WC categories in males. Table 5 suggests that greater CEA concentration (P for trend <0.01) and CEA mass (P for trend <0.01) were significantly related to menopause in women.

**Table 3:** The trend of plasma volume, CEA concentration and CEA mass by BMI category in each gender.

		BMI category (kg/m^2^)^†^				P for trend
		Less than 18.5kg/m^2^	18.5-less than 23.0kg/m^2^	23.0-less than 27.5kg/m^2^	27.5kg/m^2^ or greater	
		Mean ± SD^¶^				
Plasma volume(L)	Male	2.64±0.17	2.86±0.17	3.04±0.18	3.30±0.22	<0.01
	Female	2.39±0.13	2.52±0.14	2.66±0.15	2.90±0.21	<0.01
CEA(ng/mL)	Male	2.16±0.32	2.02±0.22	1.99±0.18	1.98±0.13	0.074
	Female	1.30±0.21	1.37±0.25	1.51±0.30	1.53±0.33	<0.01
CEA mass(μg)	Male	5.68±0.78	5.76±0.53	6.04±0.47	6.53±0.42	<0.01
	Female	3.10±0.46	3.45±0.57	3.99±0.73	4.38±0.85	<0.01

BMI, body mass index; SD, standard deviation; CEA, carcinoembryonic antigen.

P-values were calculated using the chi-square test.

^†^Stratified by the WHO recommendations for Asian population for international comparison.

^¶^Geometric means in CEA and CEA mass, adjusted for age, ALT, AST, creatinine, body fat percentage, BMI, and WC.

**Table 4:** The trend of CEA concentration and CEA mass by WC criteria in each gender.

		Waist circumference criteria (cm)^†^		P for trend
		<Criteria	≥Criteria	
		Mean ± SD^¶^		
CEA(ng/mL)	Male	1.99±0.19	2.00±0.17	0.595
	Female	1.36±0.23	1.52±0.32	<0.01
CEA mass(μg)	Male	5.85±0.46	6.41±0.45	<0.01
	Female	3.39±0.54	4.07±0.78	<0.01

SD, standard deviation; CEA, carcinoembryonic antigen.

P-values were calculated using the chi-square test.

^†^Stratified by diagnostic criteria of metabolic syndrome by National Cholesterol Education Program (NCEP) Adult Treatment Panel III (ATP III) waist circumference was divided into two groups based on 80cm for female and 90cm for male.

^¶^Geometric means adjusted for age, ALT, AST, creatinine, body fat percentage, BMI, and WC.

**Table 5:** The trend of CEA concentration and CEA mass by menopause in women.

	Menopause (≥51)		P for trend
	Pre-menopause	Post-menopause	
	Mean ± SD^¶^		
CEA(ng/mL)	1.27±0.13	1.82±0.16	<0.01
CEA mass(μg)	3.26±0.37	4.65±0.45	<0.01

SD, standard deviation; CEA, carcinoembryonic antigen.

P-values are calculated using the chi-square test.

^¶^Geometric means adjusted for age, ALT, AST, creatinine, body fat percentage, BMI, and WC.

## Discussion

Our cross-sectional study demonstrated that as visceral fat increases, serum CEA mass increases in Korean females and males. This study has once again showed that the hemodilution effect of CEA with increasing BMI occurs in only men but not in women [9].

Many previous papers have reported the hemodilution effect of tumor markers in men and women from many countries [6–8]. However, our previous study did not show the hemodilution effect of CEA in females [9]. We thought that this phenomenon was caused by visceral fat. As visceral fat measured on abdominal computed tomography (CT) increases, the serum concentration of CEA increases in women [12]. Thus, we wanted to identify the relationship between CEA concentration, CEA mass, and abdominal circumference, which represents visceral fat [19].

CEA is used to monitor disease recurrence and therapeutic efficacy in colorectal cancer and gynecologic cancer such as endometrial cancer, ovarian cancer, cervical cancer, and vulvar cancer [20–26]. However, serum CEA concentration is also mildly elevated in several nonmalignant conditions, including metabolic disturbances such as carotid atherosclerosis and metabolic syndrome, which make it difficult to monitor cancer patients [10, 11]. Thus, it is important to precisely identify the factors that affect serum CEA concentration.

From our results, as WC increases, serum CEA mass increases in both genders. It is not yet clear how visceral fat affects serum CEA mass. But, previous paper suggests that the inflammatory conditions created by increased visceral fat indirectly or directly promotes CEA production [12]. They noted that increased visceral fat resulted in increased secretion of cytokines and adipokines leading to chronic low-grade inflammatory status. Since serum CEA levels are related with a variety of chronic inflammatory disease [27, 28], increased inflammatory cytokines and adipokines due to visceral obesity may stimulate the cellular expression of CEA. Also, It has been reported that CEA can be secreted from non-CEA producing cells under certain conditions [29, 30]. For these reasons, they speculated that the altered environment due to visceral obesity would increase serum CEA levels. Basic experimental studies are needed to elucidate the precise mechanistic association between CEA and visceral obesity.

In our results, the LBM of males is significantly larger than that of females, and the body fat percentage of females is higher than that of males. It can be inferred that the proportion of bones and muscles in men is higher than in women. Bone density is 1.85g/cm^3^, fat density is 0.9g/cm^3^, and muscle density is 1.0597g/cm^3^ [31–33]. Fat is less dense than muscle and bone. Because plasma volume is determined by BSA, which is positively correlated with body weight, the amount of plasma that is increased by bones or muscles may be larger than that increased by fat. Taken together, it is also assumed that men with the same BMI have higher plasma volume than women. Also, Zhu et al. reported that the visceral fat (represented by trunk-to-limb fat mass ratio) of females is greater than that of males with a similar BMI range [34]. With similar BMI, increase of CEA mass due to visceral fat is thought to be greater in women than in men. Therefore, the hemodilution effect of CEA does not appear in females because the increased plasma volume according to BMI in females is smaller than that of males. The increased CEA mass due to visceral fat in females is higher than that of males with a similar BMI.

We also compared the CEA concentration and CEA mass of women before and after menopause. Increased visceral fat in women after menopause has already been proven several times [35, 36]. Based on this fact, we found that postmenopausal women had greater CEA concentration and CEA mass than premenopausal women. This is a reaffirmation of the effect of visceral fat on serum CEA.

This study had several limitations. First, we could not exclude the effect of confounding factors that affect the CEA levels such as smoking, hormonal levels, and past medical history. To minimize the effect of confounding factors, subjects with high serum CEA concentrations above the normal range were excluded. We also could not exclude the potential effect of racial difference. Further studies are needed to clarify the effect of visceral fat on CEA in well-organized cohort.

In conclusion, our study showed that visceral fat has an effect on increasing serum CEA mass in Korean women and men. These findings suggest that the effect of visceral fat could be considered in cancer screening using serum CEA concentration. The effect of visceral fat might also be considered when using serum CEA concentration to monitor cancer patients. This conclusion requires confirmation through large-scale studies with biochemical support.

## Supporting information

S1 Fig Receiver operating characteristics (ROC) curve for ability of the serum CEA concentration and CEA mass to identify abdominal obesity. Areas under the ROC curve were 0.522 for CEA concentration and 0.556 for CEA mass.
(TIF)

S1 Table Linear regression analysis of serum CEA concentration in female and male subjects. SE, standard error; CI, confidence interval; AST, aspartate aminotransferase; ALT, alanine aminotransferase; BMI, body mass index. P<0.001, adjusted R2 = 0.109, and Durbin-Watson = 1.966 in female subjects. P<0.001, adjusted R2 = 0.032, and Durbin-Watson = 1.984 in male subjects.
(DOCX)

S2 Table Linear regression analysis of serum CEA mass in female and male subjects. SE, standard error; CI, confidence interval; AST, aspartate aminotransferase; ALT, alanine aminotransferase; BMI, body mass index. P<0.001, adjusted R^2^ = 0.110, and Durbin-Watson = 1.962 in female subjects. P<0.001, adjusted R^2^ = 0.032, and Durbin-Watson = 1.988 in male subjects.
(DOCX)

## References

[1] Forouzanfar MH, Afshin A, Alexander LT, Anderson HR, Bhutta ZA, Biryukov S, et al Global, regional, and national comparative risk assessment of 79 behavioural, environmental and occupational, and metabolic risks or clusters of risks, 1990–2015: a systematic analysis for the Global Burden of Disease Study 2015. The Lancet. 2016;388(10053):1659–724. 10.1016/S0140-6736(16)31679-8 PMC5388856 27733284

[2] Calle EE, Kaaks R. Overweight, obesity and cancer: epidemiological evidence and proposed mechanisms. Nature Reviews Cancer. 2004;4:579 10.1038/nrc1408 15286738

[3] Olsen CM, Green AC, Whiteman DC, Sadeghi S, Kolahdooz F, Webb PM. Obesity and the risk of epithelial ovarian cancer: A systematic review and meta-analysis. European Journal of Cancer. 2007;43(4):690–709. 10.1016/j.ejca.2006.11.010 17223544

[4] Song Y-M, Sung J, Ha M. Obesity and Risk of Cancer in Postmenopausal Korean Women. Journal of Clinical Oncology. 2008;26(20):3395–402. 10.1200/JCO.2007.15.7867 18612154

[5] Esposito K., Chiodini P., Colao A., Lenzi A., Giugliano D. Metabolic syndrome and risk of cancer: a systematic review and meta-analysis. Diabetes Care. 2012;35(11):2402–2411. 10.2337/dc12-0336 23093685 PMC3476894

[6] Banez LL, Hamilton RJ, Partin AW, Vollmer RT, Sun L, Rodriguez C, et al Obesity-related plasma hemodilution and PSA concentration among men with prostate cancer. JAMA. 2007;298(19):2275–80. 10.1001/jama.298.19.2275 18029831

[7] Chang IH, Ahn SH, Han JH, Kim T-H, Kim YS, Myung SC. The Clinical Significance in Healthy Men of the Association Between Obesity Related Plasma Hemodilution and Tumor Marker Concentration. The Journal of Urology. 2009;181(2):567–73. 10.1016/j.juro.2008.10.030 19084848

[8] Werny DM, Thompson T, Saraiya M, Freedman D, Kottiri BJ, German RR, et al Obesity is negatively associated with prostate-specific antigen in U.S. men, 2001–2004. Cancer Epidemiol Biomarkers Prev. 2007;16(1):70–6. 10.1158/1055-9965.EPI-06-0588 17179487

[9] Park M, Chang IH, Kang H, Han SS. Effect of obesity-related plasma hemodilution on serum tumor marker concentration in women. J Obstet Gynaecol Res. 2015;41(5):784–9. 10.1111/jog.12621 25421332

[10] Ishizaka N, Ishizaka Y, Toda E, Koike K, Yamakado M, Nagai R. Are serum carcinoembryonic antigen levels associated with carotid atherosclerosis in Japanese men? Arterioscler Thromb Vasc Biol. 2008;28(1):160–5. 10.1161/ATVBAHA.107.155465 17951321

[11] Lee J-W, Park K-D, Im J-A, Hwang H-J, Kim S-H. Serum carcinoembryonic antigen is associated with metabolic syndrome in female Korean non-smokers. Clinica Chimica Acta. 2011;412(7):527–30. 10.1016/j.cca.2010.11.033 21138741

[12] Lee JY, Lee HK, Lee DC, Lee JW. Serum carcinoembryonic antigen is associated with abdominal visceral fat accumulation in female Korean nonsmokers. PLoS One. 2012;7(8):e43518 10.1371/journal.pone.0043518 22952699 PMC3428363

[13] Gold EB. The timing of the age at which natural menopause occurs. Obstet Gynecol Clin North Am. 2011;38(3):425–40. 10.1016/j.ogc.2011.05.002 21961711 PMC3285482

[14] Park CY, Lim JY, Park HY. Age at natural menopause in Koreans: secular trends and influences thereon. Menopause (New York, NY). 2018;25(4):423–9. 10.1097/GME.0000000000001019 29112598

[15] Shin YJ, Song JY, Kim MJ, Choi JI, Han K-D, Lee HN. Relationship between age at last delivery and age at menopause: The Korea National Health and Nutrition Examination Survey. Obstet Gynecol Sci. 2017;60(4):362–8. 10.5468/ogs.2017.60.4.362 28791268 PMC5547084

[16] Du Bois D, Du Bois EF. A formula to estimate the approximate surface area if height and weight be known. 1916. Nutrition. 1989;5(5):303–11; discussion 12–3. 2520314

[17] Boer P. Estimated lean body mass as an index for normalization of body fluid volumes in humans. American Journal of Physiology-Renal Physiology. 1984;247(4):F632–F6. 10.1152/ajprenal.1984.247.4.F632 6496691

[18] Hume R. Prediction of lean body mass from height and weight. Journal of clinical pathology. 1966;19(4):389–91. 10.1136/jcp.19.4.389 5929341 PMC473290

[19] Fujikawa R, Ito C, Mitama A. Association between visceral fat area and waist circumference measured at different sites. Diabetology International. 2012;3(3):140–5.

[20] Askalani A., Meabid H., El Sadek Saad El Sadek M., El Tabbakh Nabil M., Osman M. Immunohistochemical localization and monoclonal antibodies quantitative measurement of carcinoembryonic antigen (CEA) in cervical neoplasia. The Internet Journal of Gynecology and Obstetrics. 2002;2(1).

[21] Bruns F, Micke O, Halek G, Schäfer U, Willich N. Carcinoembryonic antigen (CEA)—a useful marker for the detection of recurrent disease in endometrial carcinoma patients. Anticancer Res. 2003;23(2A):1103–6. 12820355

[22] Duffy MJ. Carcinoembryonic antigen as a marker for colorectal cancer: is it clinically useful? Clinical chemistry. 2001;47(4):624–30. 11274010

[23] Grotowski M. [Antigens (CEA and CA 19–9) in diagnosis and prognosis colorectal cancer]. Pol Merkur Lekarski. 2002;12(67):77–80. 11957811

[24] Mitchell EP. Role of carcinoembryonic antigen in the management of advanced colorectal cancer. Semin Oncol. 1998;25(5 Suppl 11):12–20. 9786312

[25] Sørensen SS, Mosgaard BJ. Combination of cancer antigen 125 and carcinoembryonic antigen can improve ovarian cancer diagnosis. Dan Med Bull. 2011;58(11):A4331 22047929

[26] Yamawaki T, Takeshima N, Shimizu Y, Teshima H, Hasumi K. Serum Levels of Squamous Cell Carcinoma Antigen and Carcinoembryonic Antigen as Tumor Markers of Vulvar Cancer. Journal of Obstetrics and Gynaecology Research. 1996;22(4):341–6. 10.1111/j.1447-0756.1996.tb00986.x 8870416

[27] Morell AR. CEA Serum Levels in Non-Neoplastic Disease. The International Journal of Biological Markers. 1992;7(3):160–6. 1431339 10.1177/172460089200700307

[28] Stevens D, Mackay I, Busselton Population Studies G. INCREASED CARCINOEMBRYONIC ANTIGEN IN HEAVY CIGARETTE SMOKERS. The Lancet. 1973;302(7840):1238–9. 10.1016/s0140-6736(73)90975-6 4128563

[29] Chakrabarty S., Tobon A., Varani J., Brattain MG. Induction of carcinoembryonic antigen secretion and modulation of protein secretion/expression and fibronectin/laminin expression in human colon carcinoma cells by transforming growth factor-beta. Cancer research. 1988;48(14):4059–4064. 3289738

[30] Greiner JW., Guadagni F., Goldstein D., et al Evidence for the elevation of serum carcinoembryonic antigen and tumor-associated glycoprotein-72 levels in patients administered interferons. Cancer research. 1991;51(16):4155–4163. 1907881

[31] Farvid MS, Ng TW, Chan DC, Barrett PH, Watts GF. Association of adiponectin and resistin with adipose tissue compartments, insulin resistance and dyslipidaemia. Diabetes Obes Metab. 2005;7(4):406–13. 10.1111/j.1463-1326.2004.00410.x 15955127

[32] Yang J, Chiou R, Ruprecht A, Vicario J, MacPhail LA, Rams TE. A new device for measuring density of jaw bones. Dentomaxillofacial Radiology. 2002;31(5):313–6. 10.1038/sj.dmfr.4600715 12203130

[33] Ward SR, Lieber RL. Density and hydration of fresh and fixed human skeletal muscle. Journal of Biomechanics. 2005;38(11):2317–20. 10.1016/j.jbiomech.2004.10.001 16154420

[34] Zhu K, Briffa K, Smith A, Mountain J, Briggs AM, Lye S, et al Gender differences in the relationships between lean body mass, fat mass and peak bone mass in young adults. Osteoporos Int. 2014;25(5):1563–70. 10.1007/s00198-014-2665-x 24647886

[35] Toth MJ., Tchernof A., Sites CK., Poehlman ET. Menopause-related changes in body fat distribution. Annals of the New York Academy of Sciences. 2000;904:502–6. 10.1111/j.1749-6632.2000.tb06506.x 10865795

[36] Janssen I, Powell LH, Kazlauskaite R, Dugan SA. Testosterone and visceral fat in midlife women: the Study of Women's Health Across the Nation (SWAN) fat patterning study. Obesity (Silver Spring, Md). 2010;18(3):604–10. 10.1038/oby.2009.251 PMC2866448 19696765

