# Supplementary material for: The effect of visceral fat on the hemodilution effect of serum carcinoembryonic antigen in Korean population
Source: PLoS One. 2019 Dec 2;14(12):e0225649. doi: 10.1371/journal.pone.0225649 (PMC6886784; doi:10.1371/journal.pone.0225649)
Supplement: S1 Table — SE, standard error; CI, confidence interval; AST, aspartate aminotransferase; ALT, alanine aminotransferase; BMI, body mass index. P<0.001, adjusted R2 = 0.109, and Durbin-Watson = 1.966 in female subjects. P<0.001, adjusted R2 = 0.032, and Durbin-Watson = 1.984 in male subjects. (DOCX) [file pone.0225649.s002.docx]

**S1. Table. Linear regression analysis of serum CEA concentration in female and male subjects.**

|  | Female | | | | Male | | | |
| --- | --- | --- | --- | --- | --- | --- | --- | --- |
|  | β-value | SE | 95% CI | P-value | β-value | SE | 95% CI | P-value |
| Age | 0.024 | 0.001 | 0.023, 0.026 | <0.001 | 0.016 | 0.001 | 0.015, 0.018 | <0.001 |
| AST | 0.006 | 0.001 | 0.003, 0.008 | <0.001 | 0.004 | 0.001 | 0.003, 0.006 | <0.001 |
| ALT | -0.002 | 0.001 | -0.004, -0.001 | 0.103 | -0.001 | 0.001 | -0.002, 0.000 | 0.031 |
| Creatinine | 0.041 | 0.049 | -0.054, 0.137 | 0.397 | -0.182 | 0.057 | -0.293, -0.070 | 0.001 |
| Body fat percentage | -0.007 | 0.002 | -0.011, -0.003 | <0.001 | -0.015 | 0.003 | -0.020, -0.010 | <0.001 |
| BMI | 0.005 | 0.005 | -0.005, 0.015 | 0.324 | 0.016 | 0.006 | 0.004, 0.028 | 0.008 |
| Waist circumference | 0.001 | 0.002 | -0.004, 0.003 | 0.807 | 0.001 | 0.002 | -0.003, 0.006 | 0.649 |

SE, standard error; CI, confidence interval; AST, aspartate aminotransferase; ALT, alanine aminotransferase; BMI, body mass index.

P<0.001, adjusted R^2^=0.109, and Durbin-Watson=1.966 in female subjects.

P<0.001, adjusted R^2^=0.032, and Durbin-Watson=1.984 in male subjects.
