# Supplementary material for: The effect of visceral fat on the hemodilution effect of serum carcinoembryonic antigen in Korean population
Source: PLoS One. 2019 Dec 2;14(12):e0225649. doi: 10.1371/journal.pone.0225649 (PMC6886784; doi:10.1371/journal.pone.0225649)
Supplement: S2 Table — SE, standard error; CI, confidence interval; AST, aspartate aminotransferase; ALT, alanine aminotransferase; BMI, body mass index. P<0.001, adjusted R2 = 0.110, and Durbin-Watson = 1.962 in female subjects. P<0.001, adjusted R2 = 0.032, and Durbin-Watson = 1.988 in male subjects. (DOCX) [file pone.0225649.s003.docx]

**S2. Table. Linear regression analysis of serum CEA mass in female and male subjects.**

|  | Female | | | | Male | | | |
| --- | --- | --- | --- | --- | --- | --- | --- | --- |
|  | β-value | SE | 95% CI | P-value | β-value | SE | 95% CI | P-value |
| Age | 0.054 | 0.002 | 0.050, 0.058 | <0.001 | 0.036 | 0.003 | 0.031, 0.041 | <0.001 |
| AST | 0.013 | 0.003 | 0.007, 0.020 | <0.001 | 0.012 | 0.003 | 0.007, 0.018 | <0.001 |
| ALT | -0.004 | 0.003 | -0.009, 0.001 | 0.165 | -0.004 | 0.002 | -0.008, -0.001 | 0.027 |
| Creatinine | 0.177 | 0.125 | -0.069, 0.422 | 0.159 | -0.445 | 0.173 | -0.785, -0.106 | 0.010 |
| Body fat percentage | -0.027 | 0.005 | -0.038, -0.017 | <0.001 | -0.070 | 0.008 | -0.085, -0.055 | <0.001 |
| BMI | 0.067 | 0.013 | 0.042, 0.092 | <0.001 | 0.102 | 0.019 | 0.066, 0.138 | <0.001 |
| Waist circumference | 0.009 | 0.004 | 0.001, 0.018 | 0.031 | 0.041 | 0.007 | 0.028, 0.055 | <0.001 |

SE, standard error; CI, confidence interval; AST, aspartate aminotransferase; ALT, alanine aminotransferase; BMI, body mass index.

P<0.001, adjusted R^2^=0.110, and Durbin-Watson=1.962 in female subjects.

P<0.001, adjusted R^2^=0.032, and Durbin-Watson=1.988 in male subjects.
